# Bioprocess decision support tool for scalable manufacture of extracellular vesicles

**DOI:** 10.1002/bit.26809

**Published:** 2018-11-08

**Authors:** Kelvin S. Ng, James A. Smith, Matthew P. McAteer, Benjamin E. Mead, Jamie Ware, Felix O. Jackson, Alison Carter, Lino Ferreira, Kim Bure, Jon A. Rowley, Brock Reeve, David A. Brindley, Jeffrey M. Karp

**Affiliations:** ^1^ Harvard‐MIT Division of Health Sciences and Technology Cambridge Massachusetts; ^2^ Division of Engineering in Medicine, Department of Medicine Brigham & Women’s Hospital, Harvard Medical School Boston MA; ^3^ Harvard Stem Cell Institute Cambridge Massachusetts; ^4^ RoosterBio Frederick Maryland; ^5^ Nuffield Department of Orthopaedics, Rheumatology and Musculoskeletal Sciences University of Oxford Oxford UK; ^6^ The Oxford‐UCL Centre for the Advancement of Sustainable Medical Innovation, University of Oxford Oxford UK; ^7^ Department of Neurology Massachusetts General Hospital, Harvard Medical School Charlestown Massachusetts; ^8^ Broad Institute of Harvard and MIT Cambridge Massachusetts; ^9^ Koch Institute for Integrative Cancer Research, MIT Cambridge Massachusetts; ^10^ University of Coimbra, Center for Neuroscience and Cell Biology Portugal; ^11^ Department of Paediatrics University of Oxford Oxford UK; ^12^ Centre for Behavioural Medicine, UCL School of Pharmacy, University College London London UK; ^13^ UCSF‐Stanford Center of Excellence in Regulatory Science and Innovation San Francisco California

**Keywords:** costs, economics, exosomes, extracellular vesicles, manufacturing, scale‐up

## Abstract

Newly recognized as natural nanocarriers that deliver biological information between cells, extracellular vesicles (EVs), including exosomes and microvesicles, provide unprecedented therapeutic opportunities. Large‐scale and cost‐effective manufacturing is imperative for EV products to meet commercial and clinical demands; successful translation requires careful decisions that minimize financial and technological risks. Here, we develop a decision support tool (DST) that computes the most cost‐effective technologies for manufacturing EVs at different scales, by examining the costs of goods associated with using published protocols. The DST identifies costs of labor and consumables during EV harvest as key cost drivers, substantiating a need for larger‐scale, higher‐throughput, and automated technologies for harvesting EVs. Importantly, we highlight a lack of appropriate technologies for meeting clinical demands, and propose a potentially cost‐effective solution. This DST can facilitate decision‐making very early on in development and be used to predict, and better manage, the risk of process changes when commercializing EV products.

## 1 INTRODUCTION

Extracellular vesicles (EVs) are physiological nanocarriers increasingly recognized as a ubiquitous mode of intercellular signaling by which distant cells can exchange membrane and cytosolic contents, including proteins and RNA (El Andaloussi, Mäger, Breakefield, & Wood, 2013). Whether as exosomes that originate from the endolysosomal compartment, or as microvesicles that bud from the plasma membrane, EVs inherit molecules and even biological functions from their parent cells: dendritic cell EVs can act as vaccines (Robbins & Morelli, 2014), tumor‐derived EVs may promote metastasis (Becker et al., 2016), and stem cell EVs are regenerative (Lamichhane et al., 2015). Additionally, EVs may be engineered to deliver exogenous small molecules and genetic material (Kanada et al., 2015; Pegtel et al., 2010). Although the notion of using EVs for therapy only started to gain traction in the mid‐2000s, EV therapy has already been tested in more than seven human studies (Fais et al., 2016; György, Hung, Breakefield, & Leonard, 2015) and at least four companies are currently developing EV therapeutics (Smith et al., 2015), indicating a rapidly emerging industry.

Successful commercialization of EVs as therapeutic or research products requires scalable and cost‐effective manufacturing. To scale up, manufacturers embark on a complex and iterative process of identifying and testing new technologies. This is especially costly for producing cell‐derived therapeutics (Kirouac & Zandstra, 2008; Rekhi et al., 2015). Furthermore, since dosage, market size, and hence product demand can vary considerably between disease applications, the most cost‐effective solution will likely vary.

The use of decision‐support tools (DSTs) that optimize process, quality, and costs in silico has been remarkably powerful at mitigating scale‐up challenges in a wide range of industries (Kodiyalam, Yang, Gu, & Tho, 2004; Schmidt, 2005), recently including the production of cell‐derived therapeutics (Rekhi et al., 2015; Tan et al., 2014), but not EVs. Systematic and modular modeling of manufacturing processes offers great insight into cost structure and allows consideration of case‐specific needs and constraints (Hassan et al., 2015; Simaria et al., 2014). By accelerating the iterative scale‐up process, DSTs can shorten time to market and facilitate earlier patient access to new therapies in a cost‐effective manner. In this study, we describe our novel framework and its application for identifying and evaluating combinations of upstream (cell expansion) and downstream (EV harvest) technologies that minimize costs of goods (COG).

## 2 RESULTS

**Figure 1:**
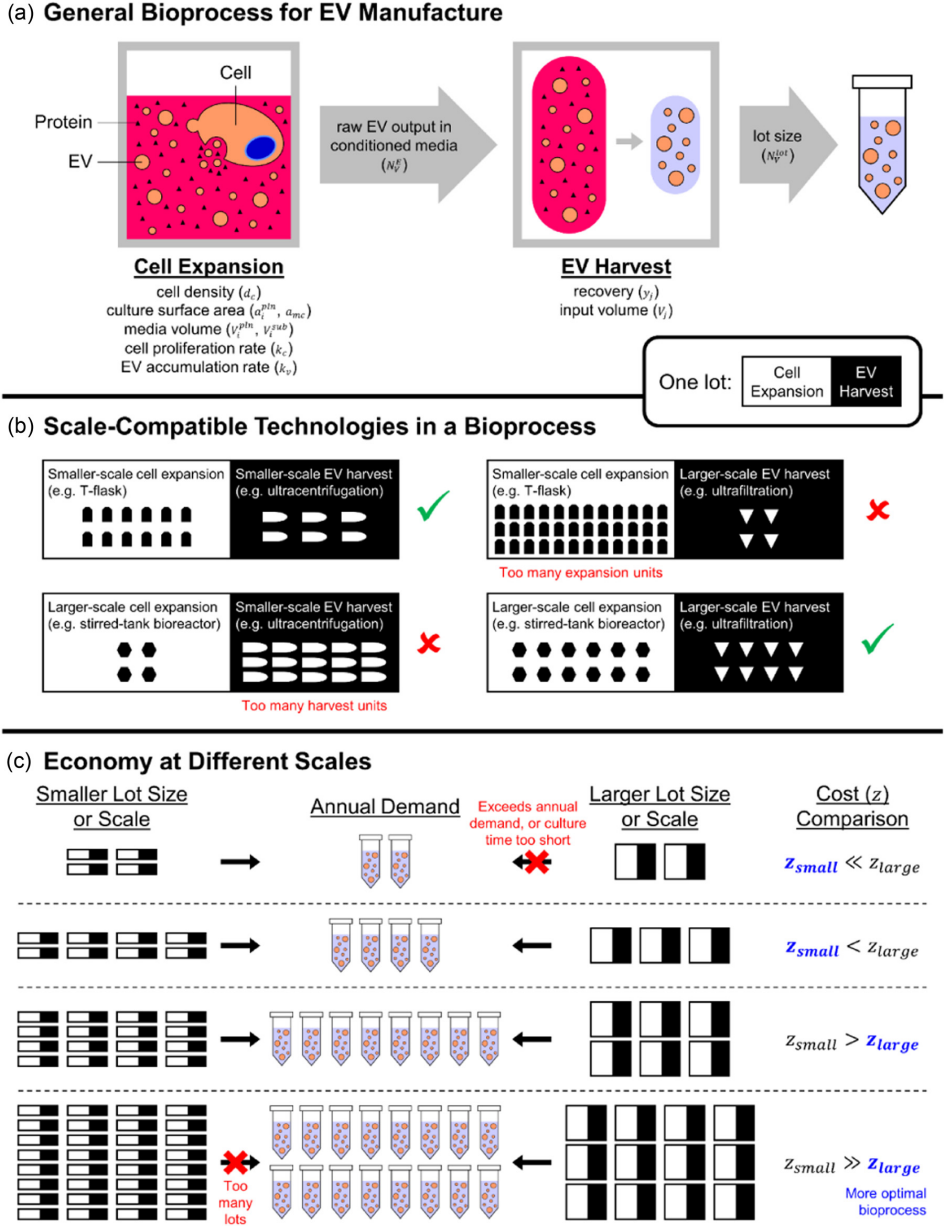
Scale, lot size, and annual demand are considered when building EV manufacturing processes for cost minimization. (a) A typical bioprocess begins with cell culture. EVs released by cultured cells accumulate in the conditioned media, which is collected for EV harvest. Some EVs are lost during harvest. One lot refers to each time a manufacturer commences cell culture and harvests EVs from the corresponding batch of cultured cells. The overall output of harvested EVs is the lot size. (b) We emphasize the difference between lot size and scale. While lot size can be arbitrarily set by the manufacturer, the scale is determined by the physical technologies used in the bioprocess. The same lot size can be met by more units of a smaller‐scale technology or fewer units of a larger‐scale technology. To account for space constraints in a facility, we set a limit to the number of units for each technology considered. (c) A given annual demand for EVs can be met by bioprocesses with different lot sizes. Bioprocesses with smaller lot sizes require more lots per year; hence the manufacturer would commence cell culture more often. In our model, we set an upper limit of 200 lots/year, so that lot sizes can be too small to meet the annual demand. Likewise, lot sizes that exceed annual demand are invalid. The same concept applies to scale: smaller‐scale bioprocesses require more units to meet the annual demand. We omit larger‐scale bioprocesses when they meet the lot size too quickly (i.e. within 24 hr upon commencement of cell culture) because they may render daily operations unfeasible. For each lot size and annual demand, we compute and compare annual costs of goods for all bioprocesses considered, and correspondingly identify the most economical technologies. In general, a smaller annual demand is most economically met using smaller‐scale technologies, and vice versa. See Supporting Information Figure 1 and Supporting Information for a detailed algorithm. EV: extracellular vesicles [Color figure can be viewed at wileyonlinelibrary.com]

EV manufacturing consists of two distinct processes: the initial cell expansion in which EVs are released into the surrounding medium, and the subsequent EV harvest from the cell culture supernatant (Figure 1). The model handles these two processes as separate modules and follows the protocol outlined in the Materials and Methods (Figure 7) to identify the most cost‐effective technology combinations for a given demand and lot size. Assumptions and limitations are presented in Box 1.

Box 1 **Assumptions and limitations imposed**

1. Cells are not harvested at the end of each lot. Users intending to harvest cells in addition to EVs can refer to studies where various cell harvest technologies are considered (Hassan et al., 2015).
2. EV harvest occurs only once per lot. In each lot, culture media introduced during seeding needs not be changed, and no new media will be added. EV harvest is the only instance when media is removed.
3. There are no EVs in the culture media when cells are initially seeded.
4. Equations and parameters governing cell proliferation and EV accumulation apply to all technologies equally.
5. To exactly match EV demand, the cell culture is terminated when just enough EVs have accumulated. This is equivalent to allowing the cells to reach the maximum allowable density and discarding extra EVs. Hence, a non‐integral $u_{E}$ value can be rounded up to the nearest integer.
6. Likewise, to exactly match volumes between cell expansion and EV harvest technologies, EV‐free buffer will be added such that a non‐integral number of EV harvest units can be rounded up to the nearest integer. Cost of this EV‐free buffer is assumed to be negligible.
7. This study also does not consider costs of pipettes, tips, common buffers, and other consumables or equipment not mentioned herein, as well as COG associated with storage, packaging, and shipping. We perform sensitivity analysis to account for uncertainty in our estimations.
8. All EV harvest technologies generate EVs of sufficient purity and usable concentration for the end application.
9. Wherever possible, disposable or single‐use technologies are considered to reflect the shift in industry preference away from hardpiped, steel‐based equipment (Gottschalk & Shukla, 2013; Langer & Rader, 2014).
10. Costs due to quality control were disregarded since release criteria for EV products are still under debate (Lener et al., 2015; Witwer et al., 2013), but this would not affect ranking between candidate technologies because the same cost of quality control would apply to all (Supporting Information Figure 4b).

### 2.1 Cell expansion

In the absence of any DSTs for modeling EV manufacturing, validation of our model was accomplished using the cell expansion module alone to reproduce cost estimates calculated previously with an allogeneic cell therapy cost modeling tool (Hassan et al., 2015; Simaria et al., 2014). Using the same parameters, our model yielded highly comparable estimates to the Simaria model (Supporting Information Section 1 and Supporting Information Figure 1).

For further validation, the cell expansion component of our model was applied to a real‐world scenario for industrial cell expansion. 10‐layer planar vessels (L‐10) are typically used (Rowley, Abraham, Campbell, Brandwein, & Oh, 2012) to produce about 2,500 doses (10^8^ cells/dose) each year, with a COG of $3.11–3.74 million (Malik, 2012). Assuming that commercial lot sizes would be at least 100 doses/lot (Brandenberger et al., 2011), our model estimated the COG using L‐10. Our estimates (Supporting Information Table 1) were shown to capture actual COG in industrial settings with reasonable accuracy. Moreover, our model shows that the industrial standard, L‐10, may not be the most economical option, and recommends larger‐scale planar vessels such as 40‐ (L‐40) and 120‐layer planar vessels (cL‐120), or even microcarrier‐based single‐use bioreactors (SUBs; Supporting Information Figure 2), as industry experts have proposed (Rowley et al., 2012).

### 2.2 Lot size, the main factor that governs the economy of scale

Application of the model to the cell expansion process permits evaluation and identification of optimal culture technologies across a range of annual demands with varied lot sizes for EV manufacturing (Figure 1b). For a given lot size, the cost structure remains largely unchanged with increasing annual demand (Supporting Information Figure 3a). Costs of consumables, labor, and quality control scale up almost linearly as the lots per year increase, whereas equipment costs are fixed. Hence, the contribution to total costs remains largely unchanged for consumables, labor, and quality control, but decreases for equipment. The number of cell expansion units remains the same as long as lot size is fixed; a comparison is made within the same set of candidate technologies since no new technologies would violate space constraints (Figure 7). With increasing annual demand, the same lot is simply repeated more times with no additional constraints unless consecutive lots begin to overlap (e.g. more than 200 lots/year; Figure 1b). Consequently, the optimal technology generally remains unchanged when lot size is fixed (Supporting Information Figure 3c).

When annual demand is fixed, increasing lot size shifts the set of valid candidate technologies from smaller‐scale to larger‐scale technologies (Supporting Information Figure 3b), and the optimal technology changes (Supporting Information Figure 3c). Smaller‐scale technologies are excluded when lot size is large enough for them to violate space constraints, while larger‐scale technologies are excluded when lot size is small enough such that the number of cells required for seeding already exceeds the lot size (Figure 1b). Using larger lot sizes to meet a given annual demand requires less labor and quality control, maximizes the capacity of culture vessels, operators, and equipment, and thereby incurs less cost.

### 2.3 Selection of EV harvest technologies

We selected EV harvest technologies (Supporting Information Table 2) from published protocols for isolating EVs from cell culture supernatants. Ultracentrifugation (UC) is the current standard for EV isolation (Gudbergsson, Johnsen, Skov, & Duroux, 2015; Smith et al., 2015; Théry, Amigorena, Raposo, & Clayton, 2006; Witwer et al., 2013). Recently marketed commercial kits include polymer‐induced precipitation (ExoQuick, System Biosciences; PPT) and size‐exclusion chromatography (qEV, IZON; SEC1; Lobb et al., 2015; Smith et al., 2015). Since the SEC1 is relatively small‐scale, taking only 0.5 ml of sample per column, we added SEC2, a size‐exclusion chromatography protocol published by an academic group that allows up to 240 ml of sample per unit (Nordin et al., 2015). Yet another emerging method is ultrafiltration. While commercial kits dedicated to EV filtration are not yet available, several academic groups have adopted commercially available filtration devices, traditionally used for isolating other forms of biological particles. UF1 represents a published protocol employing a series of dead‐end and tangential‐flow filters (Heinemann et al., 2014). Recognizing that the tangential‐flow filters can take 20 times more volume than what was published, we added UF2, a slightly modified version of UF1 to match the same tangential‐flow filter with larger‐volume dead‐end filters of the same brand, without changing any other parameters in the protocol. In general, larger‐scale versions of each method may be possible with continuous‐flow ultracentrifuges (e.g. 8 L; Hahn et al., 2013), continuous chromatography (e.g. 16 L; Bisschops, Frick, & Levison, 2016), and other industrial equipment, but feasibility of their use for EVs has not been reported.

### 2.4 Whole EV bioprocess modeling

We experimentally determined the relationship between cell and EV numbers (Methods), with a reasonable *R* ^2^ of ~0.8 (Supporting Information Table 3). A mathematically equivalent equation was previously described for bacteria (Biller et al., 2014). Primary human mesenchymal stem cells were chosen as the model cell type since their EVs have demonstrated therapeutic efficacy in more than 20 disease models (Fais et al., 2016). Cells from four different donors, cultured in StemPro serum‐free media (Gibco, Waltham, MA), were examined. Since three out of four donors behaved similarly, we used a doubling time of 22 hr and an EV output of 11,500 per cell per doubling for our model.

**Figure 2:**
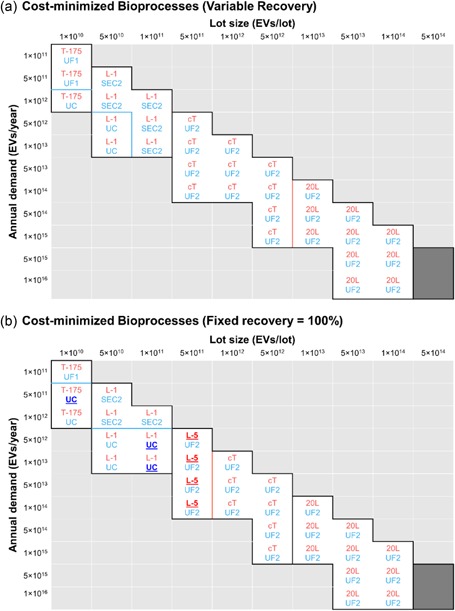
Ultrafiltration dominates as the optimal EV harvest technology in most conditions considered. Optimization is based on the total COG of every possible combination of cell expansion and EV harvest technologies. Corresponding solutions at the top and bottom halves of each cell indicate the optimal pair of (red) cell expansion and (blue) EV harvest technologies for a given lot size and annual demand. Red, blue, and black borders respectively indicate a switch in cell expansion technologies only, EV harvest technologies only, or both. Solution space that is empty either falls below 10 lots/year or exceeds 200 lots/year. Dark shaded space indicates conditions where no technology can meet within the limitations imposed. Shown are solutions when EV recovery is assumed to (a) vary between EV harvest technologies, or (b) be fixed at 100% for all EV harvest technologies. The key outcome of the latter assumption is that a given EV demand and lot size will now require the same number of units of cell expansion technology regardless of the EV harvest technology; hence, the comparison is chiefly made between EV harvest technologies, rather than whole bioprocesses. Solutions that differ between the two assumptions are underlined, emboldened, and highlighted in more contrasting colors. COG: costs of goods; EV: extracellular vesicles [Color figure can be viewed at wileyonlinelibrary.com]

Optimal cell expansion technologies for a range of EV demands span both planar vessels and SUBs, indicating that the range of lot sizes and annual demands considered was sufficiently extensive (Figure 2). Although cell numbers required to generate EVs are within the range of cell numbers in bioprocesses solely for cell expansion, solutions differ because cell expansion technologies for EV harvest are additionally limited by volume constraints: candidate technologies producing the same number of cells or EVs can significantly differ in media consumption, and require dramatically different numbers of EV harvest units.

**Figure 3:**
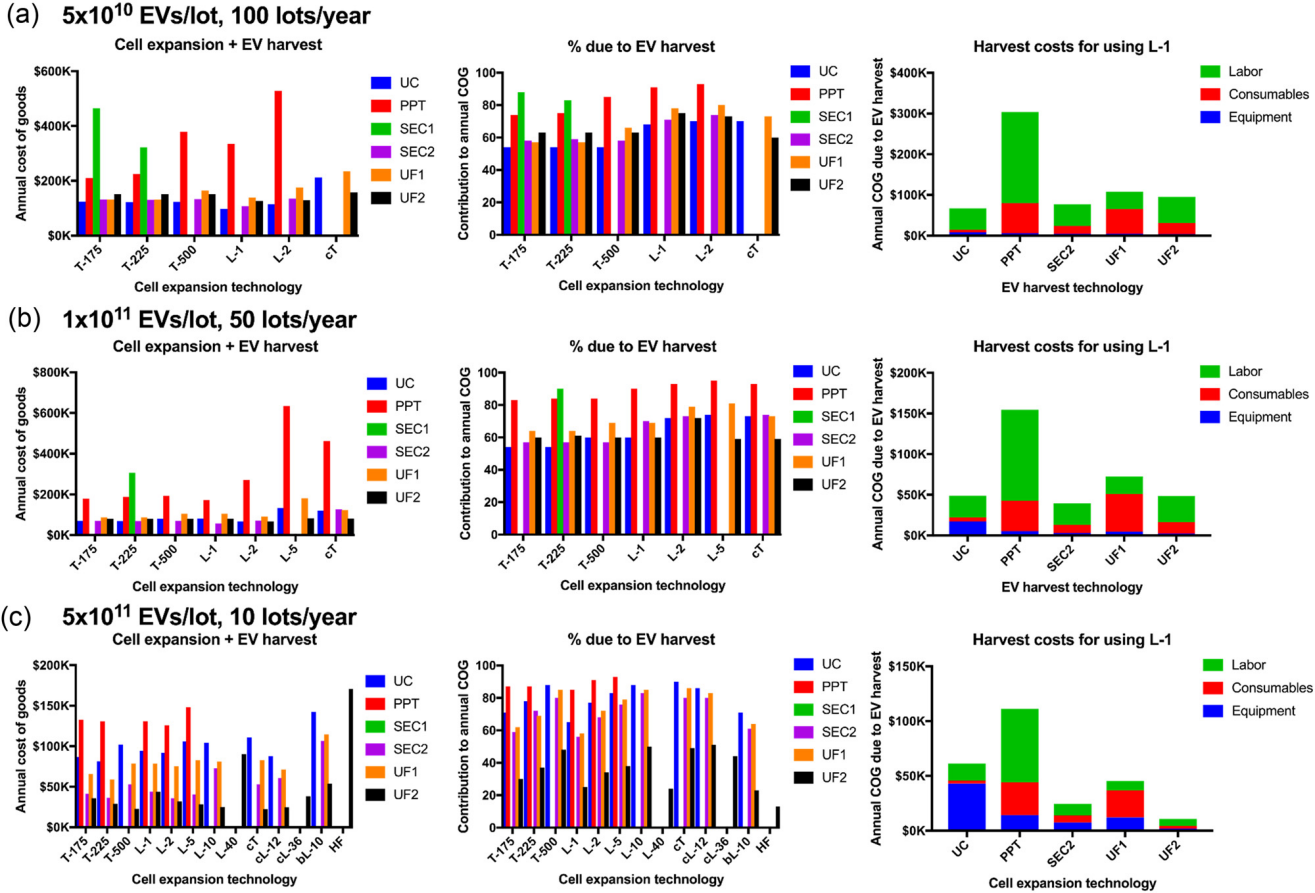
Ultrafiltration remains most cost‐effective over a large range of lot sizes. Shown are COG over a range of lot sizes for the same annual demand of 5 × 10^12^ EVs/year: (a) 5 × 10^10^ EVs/lot at 100 lots/year, (b) 1 × 10^11^ EVs/lot at 50 lots/year, and (c) 5 × 10^11^ EVs/lot at 10 lots/year. Left column, the annual cost of goods for the whole EV bioprocess for each of the suitable cell expansion and EV harvesting technologies. Middle column, the percentage contribution to the annual cost of goods due to EV harvest for each of the suitable cell expansion and EV harvesting technologies. Right column, the annual EV harvest cost when using 1‐layer planar vessels (L‐1), divided into its constituent parts (equipment, consumables and labor costs). As lot size increases, larger‐scale cell expansion technologies become valid, for which only larger‐scale EV harvest technologies can match. COG: costs of goods; EV: extracellular vesicles [Color figure can be viewed at wileyonlinelibrary.com]

Likewise, volume constraints favor EV harvest technologies that accept larger volumes, since EV‐free buffer can be added to conditioned media to meet the minimum volume of any EV harvest technology, increasing the range of lot sizes that larger‐volume technologies can tolerate without violating space constraints (Figure 3). Smaller‐volume technologies, namely PPT and SEC1, require many units, which quickly drives up costs of consumables and labor (Supporting Information Figure 4). Meanwhile, despite differing in percent recovery, UC, SEC2, and UF2 yield comparable COG in most conditions. Even when percent recovery is set at 100% for all technologies, solutions remain largely the same (Supporting Information Figure 5). This is because a smaller percent recovery needs not translate to requiring more units; a longer culture time in the same number of vessels will suffice to produce more EVs, which accumulate exponentially.

Among all methods of EV isolation considered in this study, ultrafiltration (UF1 and UF2) remain valid for the widest range of lot sizes. While they may not be optimal for all conditions considered, their COG stays close to that of the optimal technology. Until more protocols utilizing larger‐volume technologies for EV isolation are published, we conclude that ultrafiltration is currently the most versatile and cost‐effective EV isolation method for scale‐up.

### 2.5 Harvest costs dominate COG but can be reduced at large lot sizes

In general, EV harvest accounts for more than 50% of annual COG. However, when lot size is sufficiently large, the need to use larger‐scale cell expansion technologies lowers the contribution of EV harvest to overall COG.

**Figure 4:**
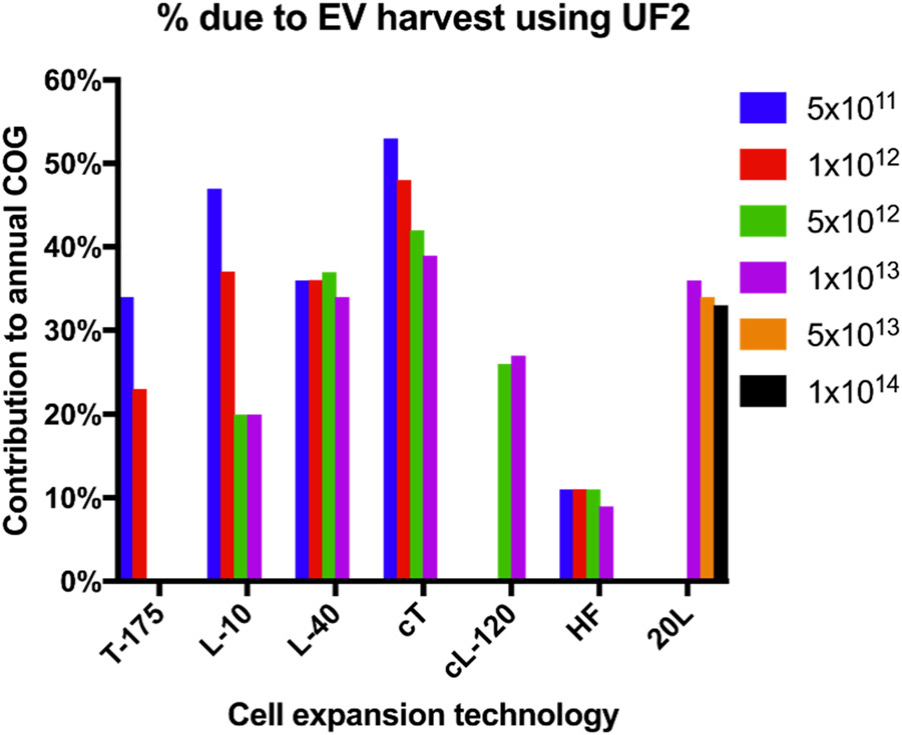
When lot size is sufficiently large, EV harvest can become cheaper than cell expansion. The legend shows different lot sizes in EVs/lot; all conditions are normalized at 100 lots/year. Tissue culture flask (T‐175), 10‐layer planar vessels (L‐10), 40‐layer planar vessels (L‐40), Compact flasks (cT), Compact multi‐layers (cL120), Hollow‐fiber bioreactors (HF), and 20 L single‐use bioreactors (20 L) were assessed to find the percentage contribution to annual COG due to ultrafiltration (UF2), for the range of lot sizes (5x10^11^– 5x10^13^ EVs/Lot). COG: costs of goods; EV: extracellular vesicles [Color figure can be viewed at wileyonlinelibrary.com]

Figure 4 shows an example using UF2. L‐10 is most widely used in the current industry (Rowley et al., 2012), while L‐40, compact flasks, cL‐120, and 20 L SUBs have been recommended for cost‐effectiveness. When coupled to these cell expansion technologies, UF2 can contribute as little as 20% to the annual COG. Meanwhile, hollow‐fiber bioreactors (HF) and SUBs are emerging cell expansion technologies being investigated for scaling up EV production (Mitchell, Court, Mason, Tabi, & Clayton, 2008; Watson et al., 2016). Because HF is relatively costly among cell expansion technologies, adding EV harvest to an HF bioprocess will consume only about 10% extra COG (Figure 4).

Trends observed in cell expansion bioprocesses (Figure 4) when varying lot size and annual demand remain largely applicable after EV harvest is incorporated. However, since the EV harvest technologies considered have not been demonstrated to be automatable, we cannot attribute the economy of scale to automation. Indeed, labor dominates in most harvest costs. Given that automation of purifying biologics is possible and being developed (Dong et al., 2016; Godawat, Konstantinov, Rohani, & Warikoo, 2015), harvest costs will likely decrease further in the future.

### 2.6 Biological parameters are the strongest cost driver

**Figure 5:**
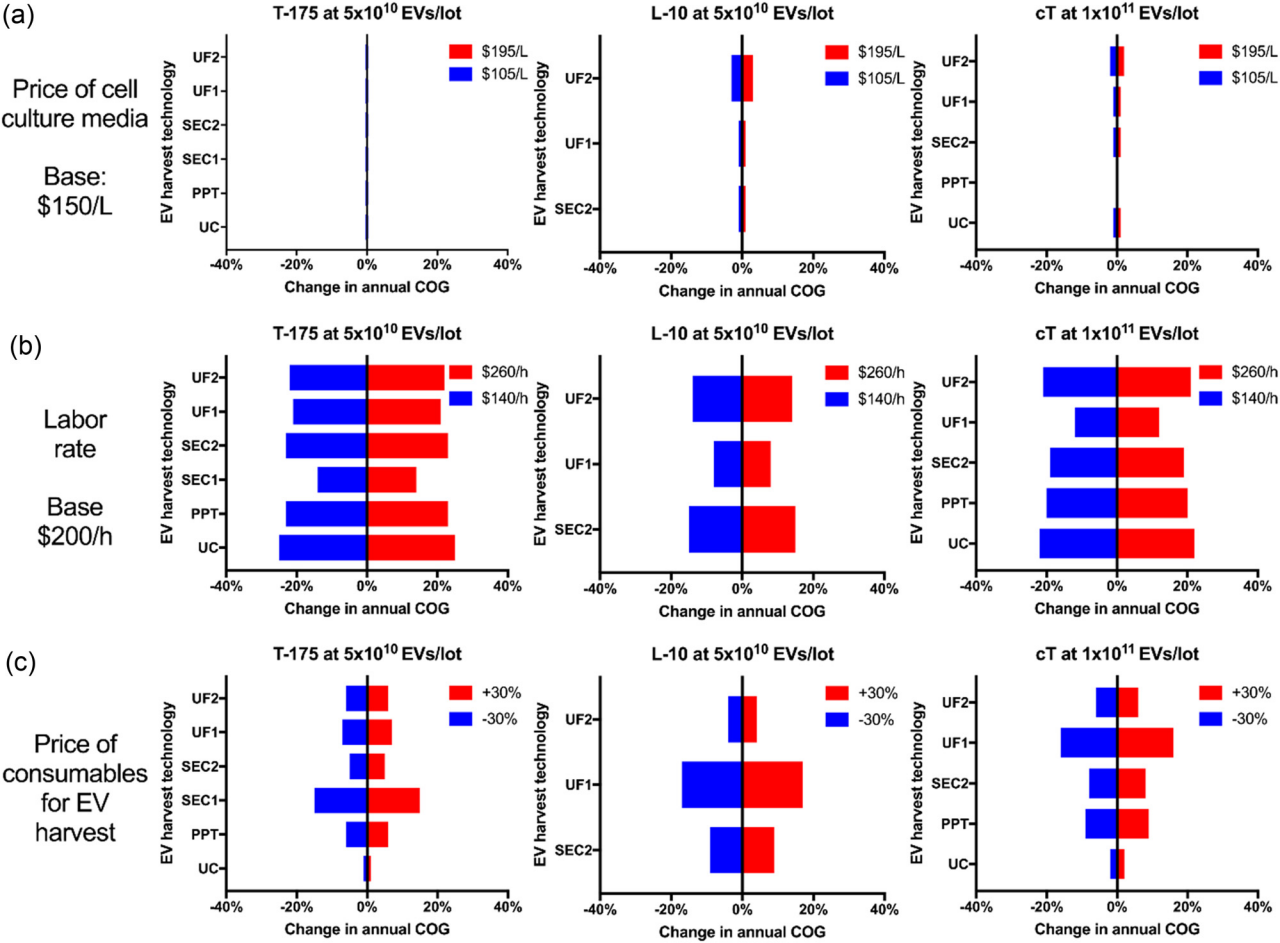
Labor rate and price of consumables for EV harvest, but not price of cell culture media, are key cost drivers. Each cost parameter is varied by ±30%, and changes in annual COG at 100 lots/year are computed for different technology combinations (Tissue culture flask [T‐175], 10‐layer planar vessels [L‐10] and Compact flasks [cT]) at different lot sizes with the selection of EV harvest technologies (Ultrafiltration [UF1 and UF2], Size‐exclusion chromatography [SEC1 and SEC2], polymer‐induced precipitation [PPT] and ultracentrifugation [UC]). (a) the “Price of cell culture media” is varied about the base price of $150/L to $195 and $105/L and the percentage change in annual COG is displayed. (b) the “Labor rate” is altered from $200 to $260 and $140/hr and the percentage change in annual COG is displayed. (c) the “price of consumables for EV harvest” is increased and decreased by 30% and the change in annual COG is displayed. COG: costs of goods; EV: extracellular vesicles [Color figure can be viewed at wileyonlinelibrary.com]

To identify key cost drivers as well as account for uncertainty in our estimates, we perform a sensitivity analysis by varying cost or process parameters and examining their individual impact on annual COG of selected technology combinations (Figure 5). Unsurprisingly, since harvest costs dominate overall COG in most conditions (Figure 3), the price of cell culture media—which concerns only cell expansion costs—hardly affects annual COG. On the contrary, labor rate and the price of consumables for EV harvest can influence annual COG almost proportionally to their degree of change. Labor rate, being involved in both cell expansion and EV harvest, is a particularly strong cost driver regardless of the technology combination, although its impact on our solutions is mild (Supporting Information Figure 5).

**Figure 6:**
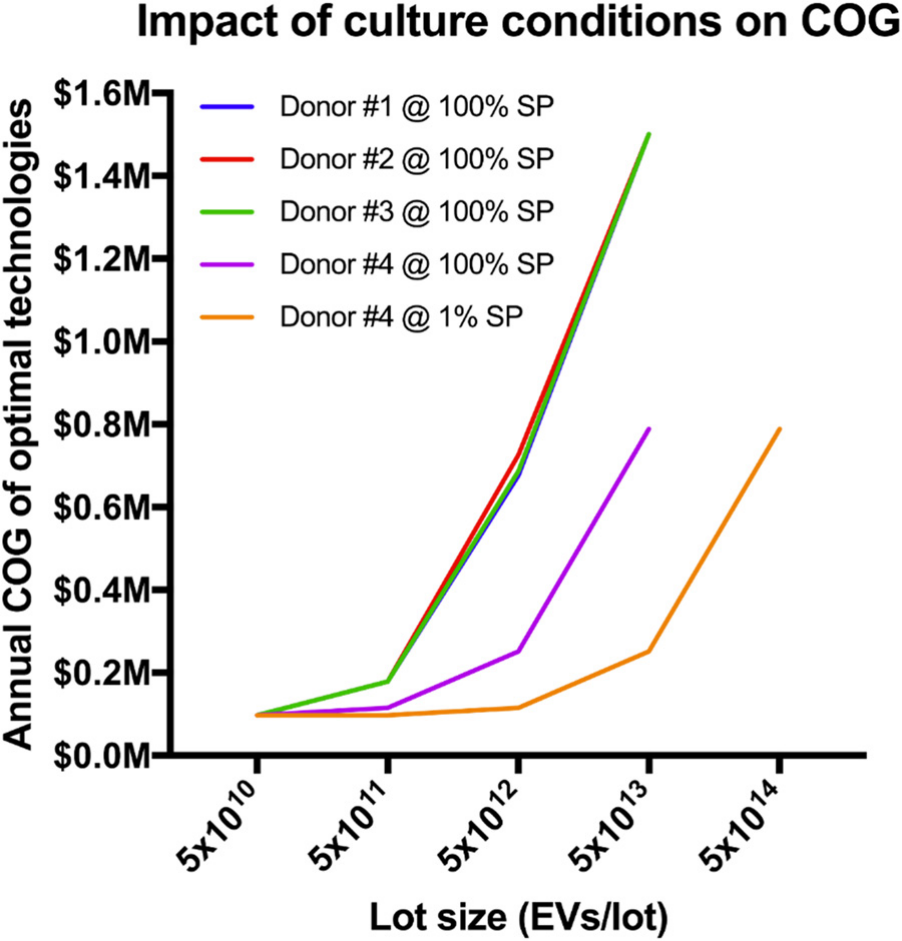
Changes in cell source or culture conditions can significantly influence COG. Biological parameters were selected from Supporting Information Table 3. Four donors’ mesenchymal stem cells were assessed for their EV output per doubling, and as donors 1–3 were similar (Supporting Information Table 3), these parameters were used for the model. Donor 4 had a significantly greater EV output per doubling: the plot shows how biological variation and culture conditions affect Annual COG at a range of lot sizes for 100 Lots/year. SP represents StemPro serum‐free medium which was used to empirically determine the biological parameters. COG: costs of goods; EV: extracellular vesicles [Color figure can be viewed at wileyonlinelibrary.com]

All computations were based on biological parameters from three donors that behaved similarly (Supporting Information Table 3). Cells from the fourth donor, which produced about three times as many EVs per population doubling, can reduce COG by more than 60% (Figure 6). Even more impressively, when cells are cultured under starvation conditions (1% of media supplement; Bian et al., 2014) that boost EV output per population doubling by an order of magnitude, COG can be reduced approximately six‐fold (Figure 6). These comparisons were based on optimal technology combinations, which could differ between biological conditions for the same lot size and annual demand. If a comparison between biological conditions was made for the same technology combination at the same lot size and annual demand, cost reduction would be more dramatic. This is because an increased EV output per population doubling means that a culture vessel produces more EVs with the same initial and final cell densities, such that EVs would be more concentrated in the conditioned media, leading to less cell expansion units, less volume, and ultimately less EV harvest units. Biological parameters are therefore the strongest cost driver.

## 3 DISCUSSION

New methods continually emerge to tackle technical and logistical challenges in EV isolation. Generally, biological effects of EV preparations have been observed regardless of the isolation method, so multiple methods could potentially be used to manufacture EVs. Due to the heterogeneity of EVs, each method typically enriches for a different EV subset with varying quality (e.g. purity, aggregation, structural integrity). Active substances and their mechanisms of action likely differ between EV products even if the products originate from the same cell source (Lener et al., 2015). Process and product become tightly intertwined; the bioprocess should be kept as consistent as possible during scale‐up to generate EV fractions with reproducible performance. This is already seen in the manufacturing of cell therapies (Campbell et al., 2015; Davie & Brindley, 2012) and glycosylated proteins (Rosenlöcher et al., 2016) such as antibodies (Ivarsson, Villiger, Morbidelli, & Soos, 2014), where small adjustments in bioprocesses can propagate to large variability in product quality. Bioprocess design should, therefore, be established as early as possible, even before empirical iterations, emphasizing the importance of DSTs. By accommodating technological variations, our model can additionally evaluate new or hypothetical methods (e.g. UF2), particularly the extent of their economic advantage over existing methods, and identify key cost drivers that may inspire and guide future innovations. Whilst our model can be adaptable to new technological developments, new insights in the field necessitate the “pressure testing” of our assumptions so that suitable updates can be made, as and when they are required. For example, switching from 2D to 3D culture will likely result in new biological parameters and media feed regimen. More advanced considerations including volume reduction and space consumption may be included. We encourage users to focus on relative comparisons of COG since absolute COG of any technology will likely change over time.

Critically, our model reveals the relative scalability between technologies, at least from an economic perspective. Scalability, historically derived from the concept of expansion flexibility, refers to the ability of a system to accommodate capacity changes without requiring significant new designs (Spice, Yip‐Hoi, & Koren, 2005). UC is generally considered not scalable. Conventional ultracentrifuges (e.g. Beckman Coulter Optima L‐90K) employ swing‐bucket or fixed‐angle rotors whose rotational axes are outside the sample chamber; to ensure a gravitational balance between the symmetrically positioned but individually and manually filled chambers, the rotors are designed to be substantially heavier than the samples. Consequently, a majority of electrical power is “wasted” on accelerating the rotor instead of the sample. At the industrial scale, such a design will warrant unrealistically large rotors and exorbitant power consumption. On the contrary, continuous‐flow ultracentrifuges (e.g. Alfa Wassermann KII) are designed so that the rotational axis is inside the sample chamber. A fluid sample will naturally balance itself symmetrically around the axis, eliminating the need to add weight to the rotor. Despite being more scalable, continuous‐flow ultracentrifuges are relatively new and have not yet been widely adopted by academic groups. Meanwhile, compared to UC, size‐exclusion chromatography (e.g. SEC1, SEC2) and ultrafiltration (e.g. UF1, UF2) are more scalable: although larger columns and filters may be necessary for industrial‐scale manufacturing, system operation and design remain the same. Perhaps the least scalable of all is polymer‐induced precipitation (e.g. PPT). The need to remove the polymer additive increases the number of steps and reliance on other purification technologies, such as centrifugation and chromatography. Solutions from our model, which incorporates both cost and bioprocess parameters in identifying optimal technologies, correlate with the relative scalability of these isolation methods.

Currently, most clinical trials investigating EV therapy are in Phase I (Fais et al., 2016; György et al., 2015), and as such, the effective dose range of EVs is unclear. We also recognize that dose will likely vary between disease applications. Hence, lot sizes and annual demand were expressed in EVs instead of doses, to allow users to define their own dose. One Phase II trial has been completed and reported, in which 22 patients were intradermally administered a median of 247 μg (by protein mass) of EVs per dose over a median of seven doses per patient (Besse et al., 2016). Given that pure EVs contain about 0.1 fg of protein each (Webber & Clayton, 2013), a median dose would consume about 2.5 × 10^12^ EVs. The maximum lot size (1 × 10^14^/lot) and annual demand (100 lots/year) we could explore in this study would supply about 4,000 of such doses per year, or treat about 570 patients a year. This already requires three units of 20 L SUBs and 15 units of UF2, or 60,000 units of UC if the current standard for EV isolation was utilized. However, we note that primary endpoints were not met in this Phase II trial. Since preclinical doses that showed efficacy in animal studies are on the order of 10^12^ EVs/kg, perhaps the effective clinical dose would be two orders of magnitude higher than what was tested in the Phase II trial. If this is true, none of the technologies investigated in this study would be feasible to meet the demand. Larger‐scale cell expansion technologies are already being investigated for scaling up EV production (Mitchell et al., 2008; Watson et al., 2016); we recommend that future studies should focus on larger‐scale EV harvest technologies capable of meeting clinical demands.

But who would be in the best position to conduct such studies? Given that the bioprocess would produce both cells and EVs, the most economical strategy may be to develop both cells and EVs as products. Our model predicts that adding EV harvest to an existing cell expansion process can cost as little as 10% of annual COG, especially if lot size is large. Therefore, current cell manufacturers may face the fewest barriers by converting conditioned media, which is otherwise regarded as waste, to commercializable products (Smith et al., 2015). EVs purified by PPT are currently sold by Systems Biosciences as “standards.” However, since PPT will unlikely be the method of choice for clinical manufacturing of EVs, “standards” generated by scalable technologies might be more appropriate. A potential disadvantage for cell manufacturers is the constraint in choice of cell culture media. If their existing bioprocesses employ EV‐containing media (e.g. serum), they would not be able to harvest EVs purely generated by the cells. Moreover, the optimal media composition for maximizing the quality and quantity of cellular products may not be optimal for producing EVs. Production of both cell and EV therapies may, therefore, be challenging; commercializing one product for research use while reserving the other for clinical use may be more realistic. By commercializing EV products generated from clinical‐grade cells, cell manufacturers would not only advance large‐scale methods for EV isolation but also provide reliable and relevant “standards” that the academic community advocates (Witwer et al., 2013).

## 4 METHODS

### 4.1 Modular modeling

EV demand is parametrized by lot size (EVs/lot) and annual demand (lots/year). One lot is defined as one stage of cell expansion followed by EV harvest. Such a modular framework allows the user to build multi‐stage bioprocesses and consider parallel processing without needing to alter the computational algorithm. Our protocol modifies a previously published model for cell manufacturing (Hassan et al., 2015; Simaria et al., 2014) to accommodate modular modeling; other published models do not specify technologies (McCall & Williams, 2013) or costs (Ungrin et al., 2012).

**Figure 7:**
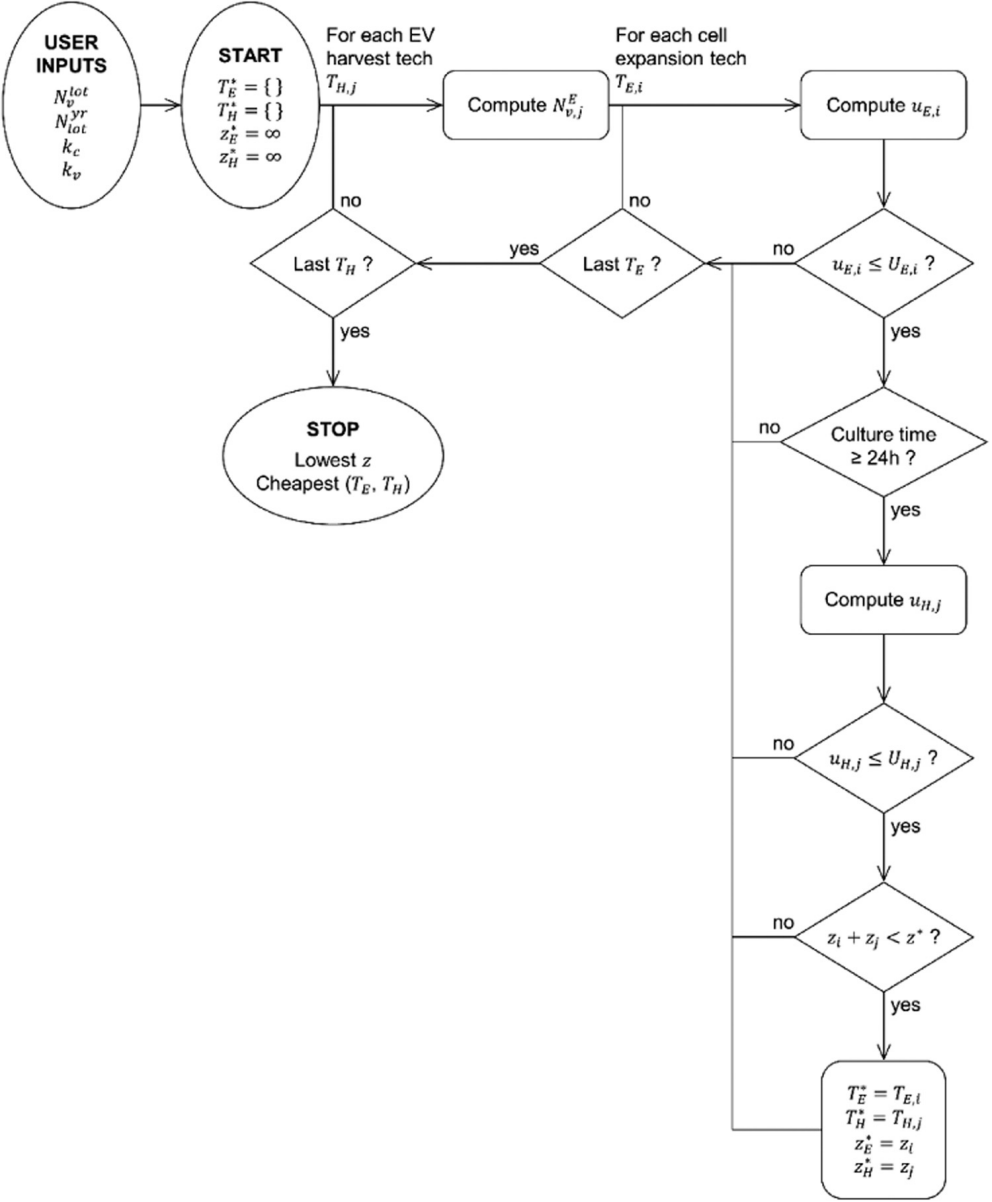
Two optimization loops compute costs and identify the cheapest combination of cell expansion and EV harvest technologies for a given demand and lot size. The inner loop optimizes for the cheapest combination for a given EV harvest technology, while the outer loop ensures that every EV harvest technology is considered. See Equations for the explanation of symbols. EV: extracellular vesicles

Figure 7 summarizes our approach. The user inputs lot size ($N_{v}^{\text{lot}}$), annual demand ($N_{\text{lot}}^{\text{yr}}$), and biological parameters ($k_{c}$, $k_{v}$) that characterize EV accumulation. We begin by using percent recovery of each EV harvest technology ($T_{H}$) to determine the number of EVs needed at the end of cell expansion before EV harvest ($N_{v}^{E}$). For a given $T_{H}$ we compute the number of units ($u_{E}$) of each cell expansion technology ($T_{E}$) and the corresponding COG ($z_{E}$). By matching the volume output of each to $T_{E}$ the volume input of the given $T_{H}$, we compute the number of units $T_{H}$ ($u_{H}$), COG for EV harvest ($z_{H}$), and the total COG (z). Recognizing space and time constraints, we impose upper limits to $u_{E}$ and $u_{H}$ ($U_{E}$, $U_{H}$) for each technology, and a minimum lag of 24 hr between cell seeding and EV harvest. Iterative comparisons of all $T_{E}$ continue for each $T_{H}$ until z is minimized and the cheapest pair of $T_{E}$ and $T_{H}$ is identified.

Box 1 lists our assumptions and limitations; Supporting Information Tables 2–5 list the numerical values used.

### 4.2 Equations

To compute the number of EVs needed at the end of cell expansion before EV harvest ($N_{v}^{E}$), divide lot size by the percent recovery ($y_{j}$) of a given $T_{H}$.

(1) $$N_{v,j}^{E}=\frac{N_{v}^{\text{lot}}}{y_{j}}$$

To compute the number of units of each cell expansion technology ($u_{E}$), first determine cell number ($N_{c}$) using cell density ($d_{c}$) and culture surface area. While a planar $T_{E}$ has a fixed surface area ($a_{i}^{\text{pln}}$), a microcarrier‐based SUB has a surface area proportional to the surface area ($a_{\text{mc}}$) and volume density ($d_{\text{mc}}$) of the microcarriers, as well as the SUB volume ($V_{i}^{\text{sub}}$).

(2) $$N_{c}=d_{c}\left(a_{i}^{\text{pln}}+a_{\text{mc}}d_{\text{mc}}V_{i}^{\text{sub}}\right)$$

The same equation can apply to all $T_{E}$ by setting $V_{i}^{\text{sub}}$ at zero for planar $T_{E}$ and $a_{i}^{\text{pln}}$ at zero for SUBs. Next, determine the maximum number of EVs ($N_{v,i}^{\max}$) that one unit of $T_{E}$ can produce, which is when cells grow from the seeding density ($d_{c}^{\min}$) to the maximum allowable density ($d_{c}^{\max}$).

(3) $$N_{v,i}^{\max}=\frac{k_{v}}{k_{c}}\left(N_{c}^{\min}-N_{c}^{\max}\right)=\frac{k_{v}}{k_{c}}\left(d_{c}^{\min}-d_{c}^{\max}\right)\left(a_{i}^{\text{pln}}+a_{\text{mc}}d_{\text{mc}}V_{i}^{\text{sub}}\right)$$

Finally, compute the number of units each $T_{E}$ needs to meet the lot size, rounded up to the nearest integer (square brackets without lower horizontal bars denote the ceiling function).

(4) $$u_{E,i}=\left\lceil \frac{N_{v,j}^{E}}{N_{v,i}^{\max}}\right\rceil$$

To compute the number of units of each EV harvest technology ($u_{H}$), divide the $T_{E}$ by the maximum sample volume ($V_{j}$) of one $T_{H}$ unit, and round up to the nearest integer. Media consumption is normalized to surface area for planar $T_{E}$ ($V_{i}^{\text{pln}}$) but not for SUBs.

(5) $$u_{H,j}=\left\lceil u_{E,i}\cdot \frac{V_{i}^{\text{pln}}a_{i}^{\text{pln}}+V_{i}^{\text{sub}}}{V_{j}}\right\rceil$$

To compute the cost of goods for the cell expansion technologies ($z_{E}$), three types of costs are considered: consumables, labor, and equipment. The cost of consumables for one lot depends on the price of vessel ($p_{i}^{\text{vess}}$), media ($p_{\text{med}}$), and microcarriers ($p_{\text{mc}}$).

(6) $$C_{i}^{\text{cons}}=u_{E,i}\left[p_{i}^{\text{vess}}+p_{\text{med}}\left(V_{i}^{\text{pln}}a_{i}^{\text{pln}}+V_{i}^{\text{sub}}\right)+{p_{\text{mc}}d}_{\text{mc}}V_{i}^{\text{sub}}\right]$$

To determine labor cost per lot, first compute the number of operators ($m_{E,i}$) needed to handle $u_{E,i}$, taking into account that each operator can manage up to $U_{E,i}^{m}$ units.

(7) $$m_{E,i}=\left\lceil \frac{u_{E,i}}{U_{E,i}^{m}}\right\rceil$$

Then compute the total wages from the hourly rate ($p_{\text{lab}}$) and the time taken to seed cells ($t_{i}^{\text{seed}}$) and collect conditioned media ($t_{i}^{\text{coll}}$). A multiplier β accounts for labor costs beyond that of an operator (e.g. supervisors and management).

(8) $$C_{i}^{\text{lab}}=m_{E,i}∙p_{\text{lab}}\left(t_{i}^{\text{seed}}+t_{i}^{\text{coll}}\right)\left(1+\beta \right)$$

Equipment for cell expansion is split into three categories: incubators where cells undergo expansion, biosafety cabinets (BSCs) where cells are handled under aseptic conditions, and ancillary equipment such as those for automation. The price of incubators ($p_{i}^{\text{inc}}$) or ancillary equipment ($p_{i}^{\text{anc}}$) depends on the specific $T_{E}$ and each incubator or ancillary equipment can process up to $U_{i}^{\text{inc}}$ or $U_{i}^{\text{anc}}$ units simultaneously. The price of a BSC ($p_{\text{bsc}}$) is the same for any expansion technology and a BSC can be used by up to $U^{\text{bsc}}$ operators at a time. A delta function ($\delta_{i}$) indicates if a particular $T_{E}$ requires a BSC. Add the three costs to compute equipment costs per lot.

(9) $$C_{i}^{\text{eq}}=p_{i}^{\text{inc}}\left\lceil \frac{u_{E,i}}{U_{i}^{\text{inc}}}\right\rceil +p_{\text{bsc}}\left\lceil \frac{m_{E,i}∙\delta_{i}}{U^{\text{bsc}}}\right\rceil +p_{i}^{\text{anc}}\left\lceil \frac{u_{E,i}}{U_{i}^{\text{anc}}}\right\rceil$$

Use $N_{\text{lot}}^{\text{yr}}$ to compute the annual COG. Since equipment can be shared between lots, an additional lot in the same year does not increase equipment cost, but equipment can depreciate over time ($t_{\text{dep}}$). Annual COG of each $T_{E}$ is stored as $z_{i}$, while the global minimum (i.e. annual COG of optimal combination of $T_{E}$ and $T_{H}$) among all values of $z_{i}$ is stored as $z_{E}$.

(10) $$z_{i}=N_{\text{lot}}^{\text{yr}}\left(C_{i}^{\text{cons}}+C_{i}^{\text{lab}}\right)+\frac{C_{i}^{\text{eq}}}{t_{\text{dep}}}$$

To compute the cost of goods for the EV harvest technologies ($z_{H}$), likewise, annual COG for EV harvest is broken down into consumables, labor, and equipment. One $T_{H}$ unit represents a multi‐step process of purification, with an overall price of consumables ($p_{j}^{\text{cons}}$). Consumables include single‐use tubes, chromatography columns, and polymers for precipitation.

(11) $$C_{j}^{\text{cons}}=u_{H,j}∙p_{j}^{\text{cons}}$$

Similarly, compute labor cost per lot for EV harvest units, but instead using the total labor time ($t_{j}^{\text{proc}}$) for the entire multi‐step process. Waiting between steps is not considered labor.

(12) $$m_{H,j}=\left\lceil \frac{u_{H,j}}{U_{H,j}^{m}}\right\rceil$$

(13) $$C_{j}^{\text{lab}}=m_{H,j}∙p_{\text{lab}}∙t_{j}^{\text{proc}}\left(1+\beta \right)$$

Equipment for EV harvest involves only BSCs and ancillary equipment such as benchtop centrifuges. Because BSCs may also be used for cell expansion, they are considered in computing $z_{H}$ only if additional BSCs are required for EV harvest. Annual COG of each $T_{H}$ is stored as $z_{j}$; the global minimum is stored as $z_{H}$.

(14) $$C_{j}^{\text{eq}}=\left\{ \begin{array}{cc} p_{j}^{\text{anc}}\left\lceil \frac{u_{H,j}}{U_{j}^{\text{anc}}}\right\rceil +p_{\text{bsc}}\left(\left\lceil \frac{m_{H,j}}{U^{\text{bsc}}}\right\rceil -\left\lceil \frac{m_{E,i}∙\delta_{i}}{U^{\text{bsc}}}\right\rceil \right) & \text{if }\left\lceil \frac{m_{H,j}}{U^{\text{bsc}}}\right\rceil >\left\lceil \frac{m_{E,i}∙\delta_{i}}{U^{\text{bsc}}}\right\rceil \\ p_{j}^{\text{anc}}\left\lceil \frac{u_{H,j}}{U_{j}^{\text{anc}}}\right\rceil & \text{if }\left\lceil \frac{m_{H,j}}{U^{\text{bsc}}}\right\rceil \leq \left\lceil \frac{m_{E,i}∙\delta_{i}}{U^{\text{bsc}}}\right\rceil \end{array}\right.$$

(15) $$z_{j}=N_{\text{lot}}^{\text{yr}}\left(C_{j}^{\text{cons}}+C_{j}^{\text{lab}}\right)\cdot \frac{C_{j}^{\text{eq}}}{t_{\text{dep}}}$$

## 4.3 Determination of biological parameters

Human mesenchymal stem cells were cultured on CELLstart substrate (Gibco, Waltham, MA USA) in StemPro serum‐free media (Gibco, Waltham, MA USA). Conditioned media were sampled at reported time points, clarified of cellular debris and apoptotic bodies by centrifugation at 500 rcf for 10 min followed by 2000 rcf for 20 min, and finally analyzed for particle concentration using nanoparticle tracking analysis (NanoSight NS300, Malvern, UK). Since cell‐free controls demonstrated that CELLstart and StemPro do not contain nor release measurable particles, all measurable particles in the conditioned media were deemed as EVs. Doubling time of cell expansion was calculated using seeding and harvest densities, and the duration between seeding and harvest (Supplementary Figure 6). When cell and EV numbers from different donors and culture conditions were fitted into Equation 3, the average R2 was 0.83 ± 0.081.

## CONFLICTS OF INTEREST

D.A. Brindley is a stockholder in Translation Ventures Ltd. (Charlbury, Oxfordshire, UK), IP Asset Ventures Ltd. (Oxford, Oxfordshire, UK), and Biolacuna Ltd., companies that, among other services, provide cell therapy biomanufacturing, regulatory, and financial advice to pharmaceutical clients. D.A. Brindley is also subject to the CFA Institute's codes, standards, and guidelines, so he must stress that this piece is provided for academic interest only and must not be construed in any way as an investment recommendation. Additionallry, at the time of publication, D.A. Brindley and the organizations with which he is affiliated may or may not have agreed and/or have pending funding commitments from the organizations named here.

## Supporting information

Supporting information
