# Supplementary material for: Bioprocess decision support tool for scalable manufacture of extracellular vesicles
Source: Biotechnol Bioeng. 2018 Nov 8;116(2):307–19. doi: 10.1002/bit.26809 (PMC6322973; doi:10.1002/bit.26809)
Supplement: Supplementary file 1 — Supporting information [file BIT-116-307-s001.docx]

**Supporting Information**

Supplementary Section 1 - *Model validation against the Simaria model*

To validate the accuracy of our model, we first attempted to reproduce cost estimates previously published for cell expansion bioprocesses, specifically the Simaria model. Unlike our model which focuses on one stage of cell expansion, the Simaria model assumes a four-stage bioprocess (i.e. four passages from P1 to P4) regardless of the demand for cells. Each stage would utilize a different cell expansion technology, and optimization would seek to minimize COG incurred throughout all four stages. The algorithm starts with P4, uses P4 parameters to pick the cheapest technology for P3, and so on until P1. Simaria *et al.* provided absolute numbers only in a case study comparing three bioprocesses at 50 doses/lot versus 1000 doses/lot at a dose size of 107 cells/dose and annual demand of 10,000 doses/year; hence we used the same conditions in the case study to validate our estimates **(Supplementary Fig. 1)**.

The Simaria model indicates that the optimal (i.e. most economical) bioprocesses to produce 50 and 1000 doses/lot would utilize L-40 **(Supplementary Fig. 1A)** and cL-120 (**Supplementary Fig. 1D)** respectively at the last stage (i.e. P4). Because annual demand is the same, a difference in lot size reflects a difference in the number of lots per year. At 50 doses/lot, the same bioprocess would be repeated 20 times more in a year than it would at 1000 doses/lot. Hence, quality control, which incurs the same cost per lot regardless of lot size, would cost more per year with a smaller lot size. Meanwhile, costs of labor and consumables are sensitive to lot size. Generally, for larger-scale technologies, consumables cost more because of higher media consumption, but labor costs less because of automation. When lot size is small, although larger-scale technologies would save labor, they would ‘waste’ media as cells need not be cultured to maximum density to meet the lot size; smaller-scale technologies therefore become more favorable despite the higher labor cost. Equipment does not cost much annually since they can be reused between lots and depreciate over time, even though the start-up cost of ancillary equipment (e.g. $425,000 for cL-120) can be significant.


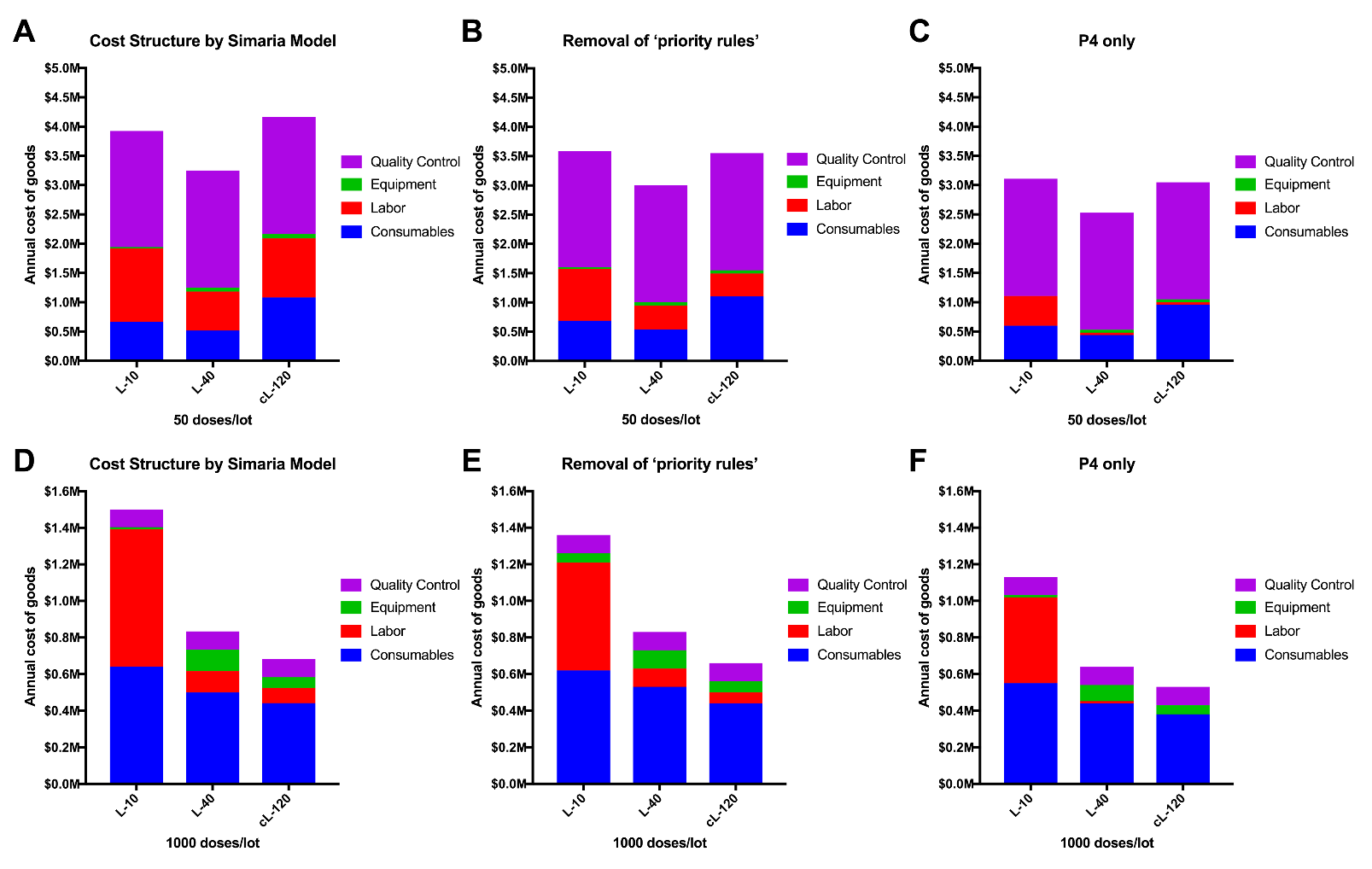


Supplementary Figure 1. Cost estimates for cell expansion change only slightly when ‘priority rules’ are removed, and are dominated by the last passage. (A) The Simaria model indicates that a four-stage bioprocess ending with L-40 is the most economical for a lot size of 50 doses/lot. (B) Removing ‘priority rules’ from the Simaria model slightly lowers cost estimates but preserves the optimal technology for P4. (C) Reducing the cell expansion bioprocess from four stages to one stage further lowers cost, and still yields the same optimal technology. (D-F) The same observations are made for a lot size of 1000 doses/lot.

|  | COG (Millions USD$) | | | |
| --- | --- | --- | --- | --- |
| Doses/lot | 1-stage | 2-stage | 3-stage | 4-stage |
| 10 | 5.27 | 5.62 | 5.75 | 5.86 |
| 25 | 3.64 | 4.05 | 4.14 | 4.19 |
| 50 | 3.07 | 3.42 | 3.48 | 3.53 |
| 100 | 2.83 | 3.14 | 3.20 | 3.23 |

Supplementary Table 1. Cost estimates (Millions USD$) for the different doses/lot using either a 1, 2, 3, or 4-stage bioprocess Costs were estimated using our model for a quantity typically produced in industrial settings (2500 doses at 10^8^ cells/dose) with the standard 10-layer planar vessels. One stage of cell expansion followed by EV harvest is defined as one lot. The model’s modular framework allows the user to build multi-stage bioprocesses and consider parallel processing without needing to alter the computational algorithm. A 1, 2, 3, and 4-stage model was employed to estimate costs for different bioprocesses, depending on how many bioprocess stages are allowed. The real world estimated cost totals between $3.11-3.74 million annually showing that our estimates capture actual costs with a reasonable degree of accuracy.


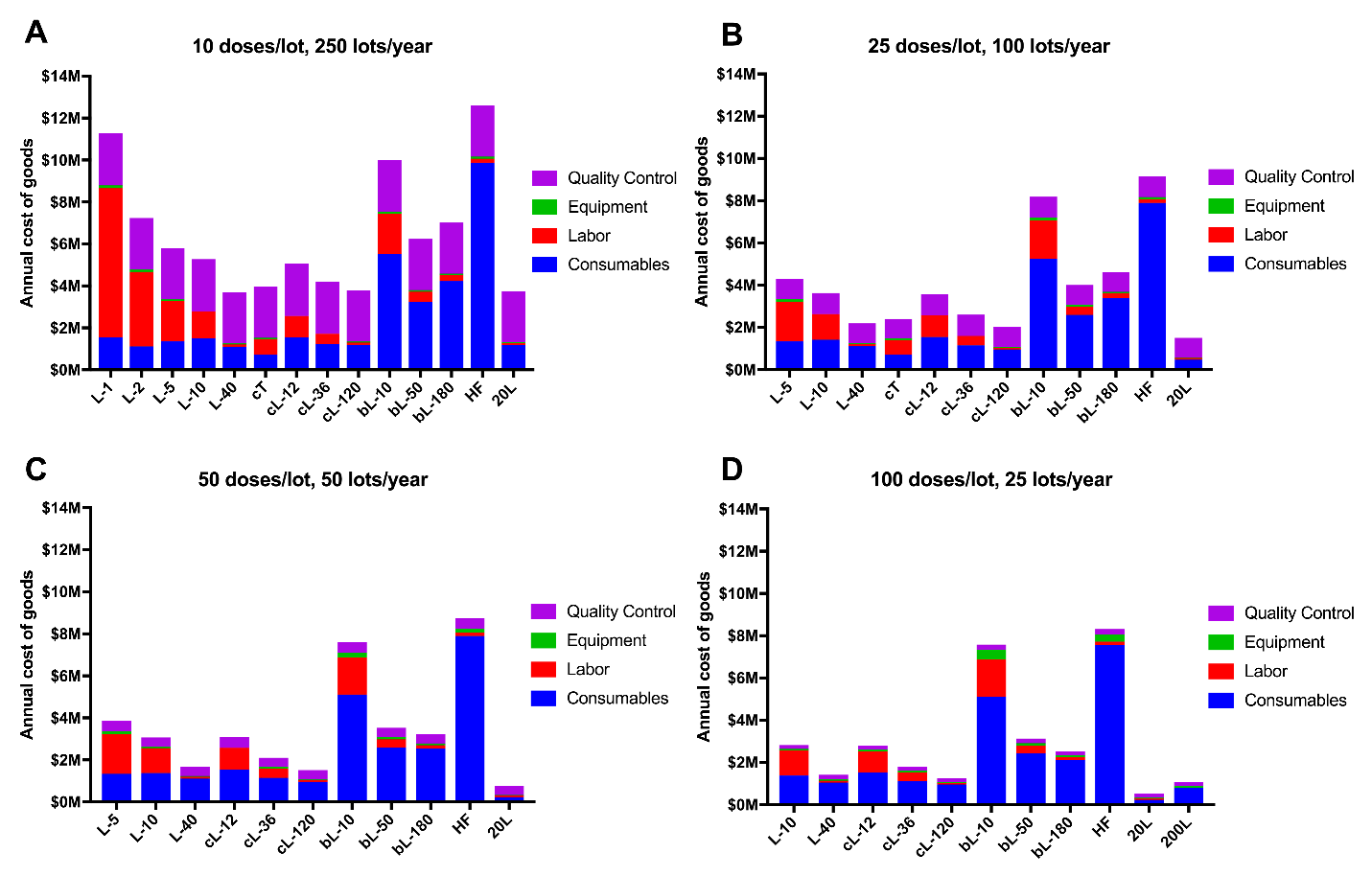


Supplementary Figure 2. Technologies (e.g. L-10) currently preferred by cell therapy industry may not be the most economical to meet market demands (2500 doses/year). Plots displaying the annual Cost of Goods (COG) for a range of different cell expansion technologies, showing the contributing different cost categories (Equipment, Consumables, Labor, and Quality Control) that lead to the overall COG. Different lot sizes (# doses/lot) were assessed with a fixed overall demand (2500 lots/year) to show it affects technology selection based on cost efficiency. Use of larger-scale planar vessels (e.g. L-40, cL-120) or SUBs (e.g. 20L, 200L) may further reduce cost of cell expansion when compared to the industry standard L-10.


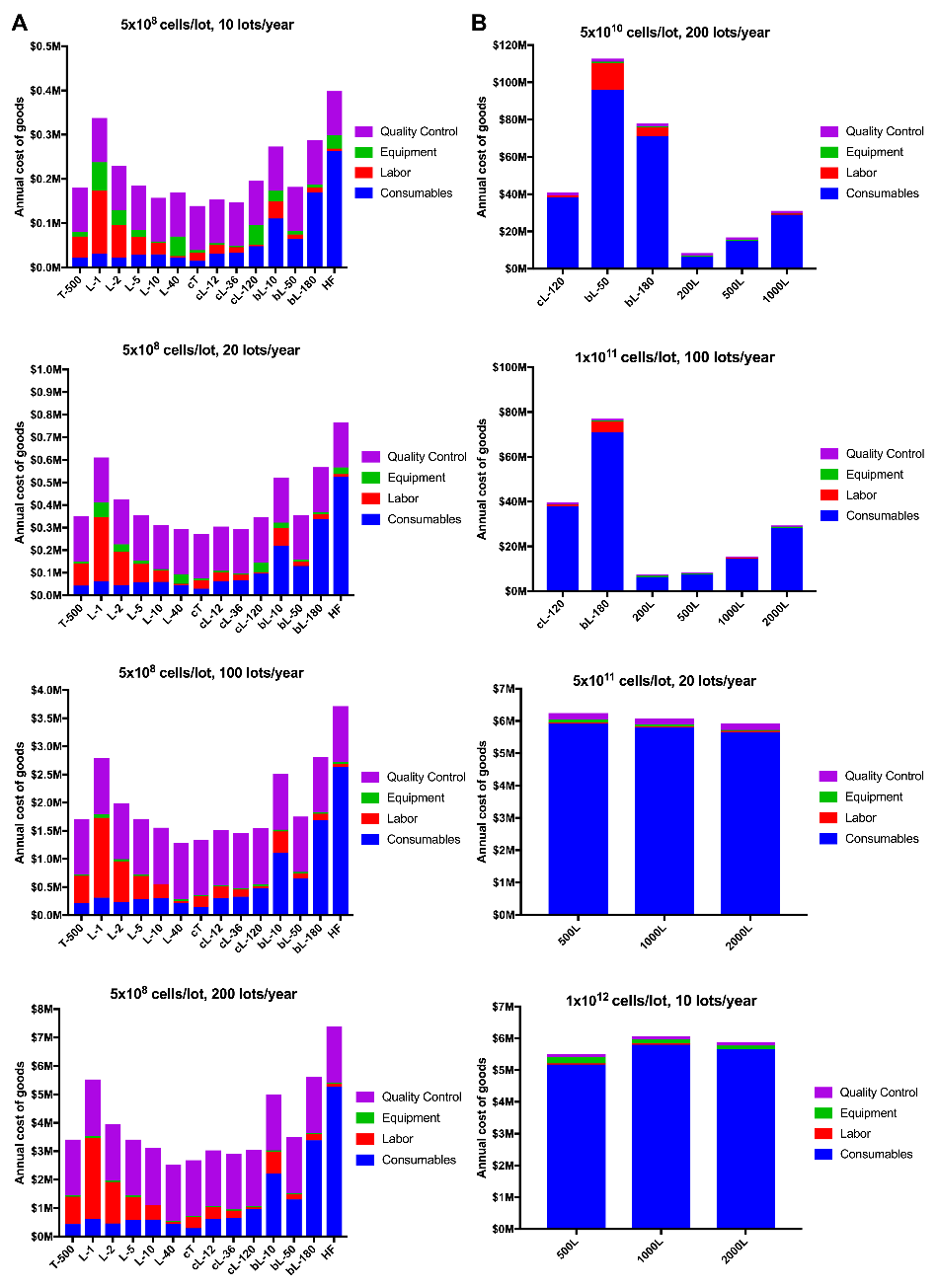


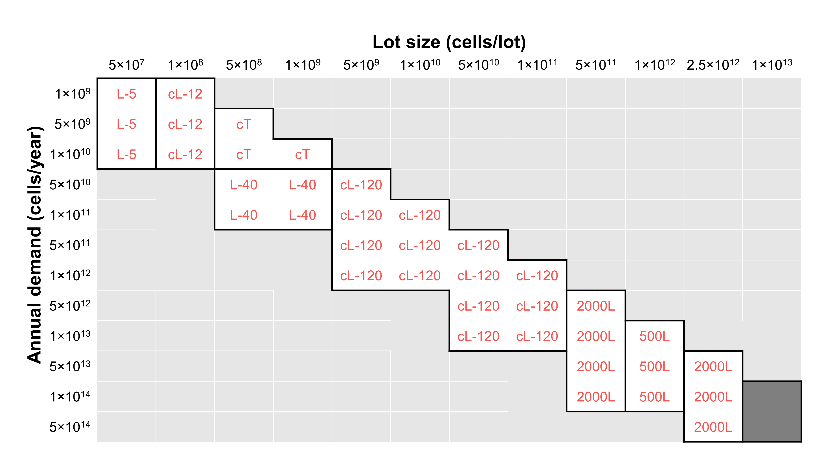


**C**

Supplementary Figure 3. Lot size, more so than annual demand, drives a switch in optimal technology for cell expansion. (A) For a fixed lot size, varying annual demand does not drastically change the optimal technology. (B) For a fixed annual demand, varying lot size quickly drives a change in scale and hence a change in optimal technology. Excluded technologies either violate space constraints, or already exceed lot size upon seeding. (C) Optimal technologies for cell expansion to meet a range of overall demand at a range of lot sizes. Solution space that is empty either falls below 10 lots/year or exceeds 200 lots/year. Shaded space indicates conditions that no technology can meet within the limitations imposed (e.g. maximum number of units). SUBs are considered only when no planar vessel can meet the conditions within space constraints.

Supplementary Table 2. Process and cost parameters for EV harvest technologies.

| EV harvest technology | | Percent recovery | Unit sample volume (mL) | Consumables price ($) | Operator capacity | Labor time (h) | Ancillary capacity | Ancillary price ($) | Maximum number of units per lot |
| --- | --- | --- | --- | --- | --- | --- | --- | --- | --- |
|  |  | $y_{j}$ | $V_{j}$ | $p_{j}^{cons}$ | $U_{H,i}^{m}$ | $t_{j}^{proc}$ | $U_{j}^{anc}$ | $p_{j}^{anc}$ | $U_{H,i}$ |
| **Ultracentrifugation** | | | | | | | | | |
| UC | *Remove cells and cell debris*   1. 6 PS tubes (10 min) → 3,000*g* in BC   *Pellet and rinse EVs*   1. 6 PA (15 min) → 100,000*g* in UC 2. 6 PA (15 min) → 100,000*g* in UC 3. Collect (10 min)   Ancillary equipment: 1 BC, 1 UC | 0.50 | 240 | 51 | 2 | 0.83 | 1 | 86,000 | 8 |
| **Polymer-induced precipitation** | | | | | | | | | |
| PPT | *Remove cells and cell debris*   1. 1 PS tube (10 min) → 3,000*g* in BC   *Precipitate EVs*   1. 1 PS tube + 2 mL EQ-TC (10 min) → 1,500*g* in BC 2. Aspirate (5 min) → 1,500*g* in BC   *Remove polymer from EVs*   1. 1 microtube + 1 spin column (60 min) → 800*g* in MC 2. 1 microtube (10 min) → 800*g* in MC 3. Collect (10 min)   Ancillary equipment: 1 BC, 1 MC | 0.80 | 10 | 46.50 | 12 | 1.75 | 12 | 18,000 | 96 |
| **Size-exclusion chromatography** | | | | | | | | | |
| SEC1 | *Remove cells and cell debris*   1. 1 microtube (5 min) → 3,000*g* in MC   *Elute EVs*   1. 1 microtube + 1 qEV column (10 min) 2. Collect (10 min)   Ancillary equipment: 1 MC | 0.90 | 0.5 | 13.50 | 30 | 0.42 | 30 | 7,000 | 240 |
| SEC2 | *Remove cells and cell debris*   1. 6 PS tubes (10 min) → 3,000*g* in BC   *Concentrate and rinse EVs*   1. 16 DEF1 (20 min) → 3,500*g* in BC   *Elute EVs*   1. 1 PS tube + 1 HiPrep column (10 min) → SEC pump 2. Collect (10 min)   Ancillary equipment: 1 BC, 1 SEC pump | 0.90 | 240 | 212 | 2 | 0.83 | 1 | 21,000 | 16 |

Supplementary Table 2 (continued). Process and cost parameters for EV harvest technologies.

| EV harvest technology | | Percent recovery | Unit sample volume (mL) | Consumables price ($) | Operator capacity | Labor time (h) | Ancillary capacity | Ancillary price ($) | Maximum number of units per lot |
| --- | --- | --- | --- | --- | --- | --- | --- | --- | --- |
|  |  | $y_{j}$ | $V_{j}$ | $p_{j}^{cons}$ | $U_{H,i}^{m}$ | $t_{j}^{proc}$ | $U_{j}^{anc}$ | $p_{j}^{anc}$ | $U_{H,i}$ |
| **Ultrafiltration** | | | | | | | | | |
| UF1 | *Remove cells and cell debris*   1. 1 DEF2 (10 min)   *Concentrate and purify EVs*   1. 1 TFF (20 min) → TFF pump 2. Collect (10 min)   Ancillary equipment: 1 TFF pump | 0.50 | 150 | 235 | 4 | 0.67 | 1 | 15,000 | 16 |
| UF2 | *Remove cells and cell debris*   1. 3 DEF3 (20 min)   *Concentrate and purify EVs*   1. 1 TFF (20 min) → TFF pump 2. Collect (20 min)   Ancillary equipment: 1 TFF pump | 0.50 | 3,000 | 297 | 4 | 1.00 | 1 | 15,000 | 16 |

| PS tubes: polystyrene tubes, $0.50 ea  PA tubes: polyallomer tubes, $4 ea  Microtubes, $0.25 ea  EQ-TC: ExoQuick for Tissue Culture, $19/mL  Spin column, $7 ea  qEV column, $65 ea, reusable up to 5 times  HiPrep column, $650 ea, reusable up to 20 times | DEF1: Amicon 15-mL centrifugal filters, $11 ea  DEF2: EMD Millipore Stericup 250-mL filters, $10 ea  DEF3: EMD Millipore Stericup 1-L filters, $24 ea  TFF: Spectrum Labs MidiKros filters, $225 ea | UC: Beckman Coulter L90k ultracentrifuge with 1 rotor, $75,000 ea  BC: Eppendorf 5804R benchtop centrifuge with 1 rotor, $11,000 ea  MC: Eppendorf 5424R microcentrifuge with 1 rotor, $7,000 ea  SEC pump: GE Healthcare ÄKTA system, $10,000 ea  TFF pump: Spectrum Labs KrosFlo with pressure monitor, $15,000 ea |
| --- | --- | --- |

Supplementary Table 3. Measured and calculated values for cell growth and EV output parameters.

| Donor | Culture medium | *R*^2^ for fit to  **Equation 3** | Doubling time (hours) | EV output per doubling, $k_{v}/k_{c}$  (EVs/cell) |
| --- | --- | --- | --- | --- |
| 1 | 100% StemPro | 0.861 | 22.1 | 11,524 |
| 2 | 100% StemPro | 0.792 | 18.9 | 9,589 |
| 3 | 100% StemPro | 0.859 | 16.2 | 11,171 |
| 4 | 100% StemPro | 0.852 | 37.7 | 38,782 |
| 4 | 1% StemPro | 0.833 | 404 | 389,947 |

The experimentally derived relationship between cell and EV numbers were determined for four donors to calculate the EV output. 100% StemPro culture medium was used for all donors, and 1% StemPro was also used for donor four to show the effect that culture medium has upon EV output. An R^2^ value was calculated for the resulting growth curves which was applied to **Equation 3.**


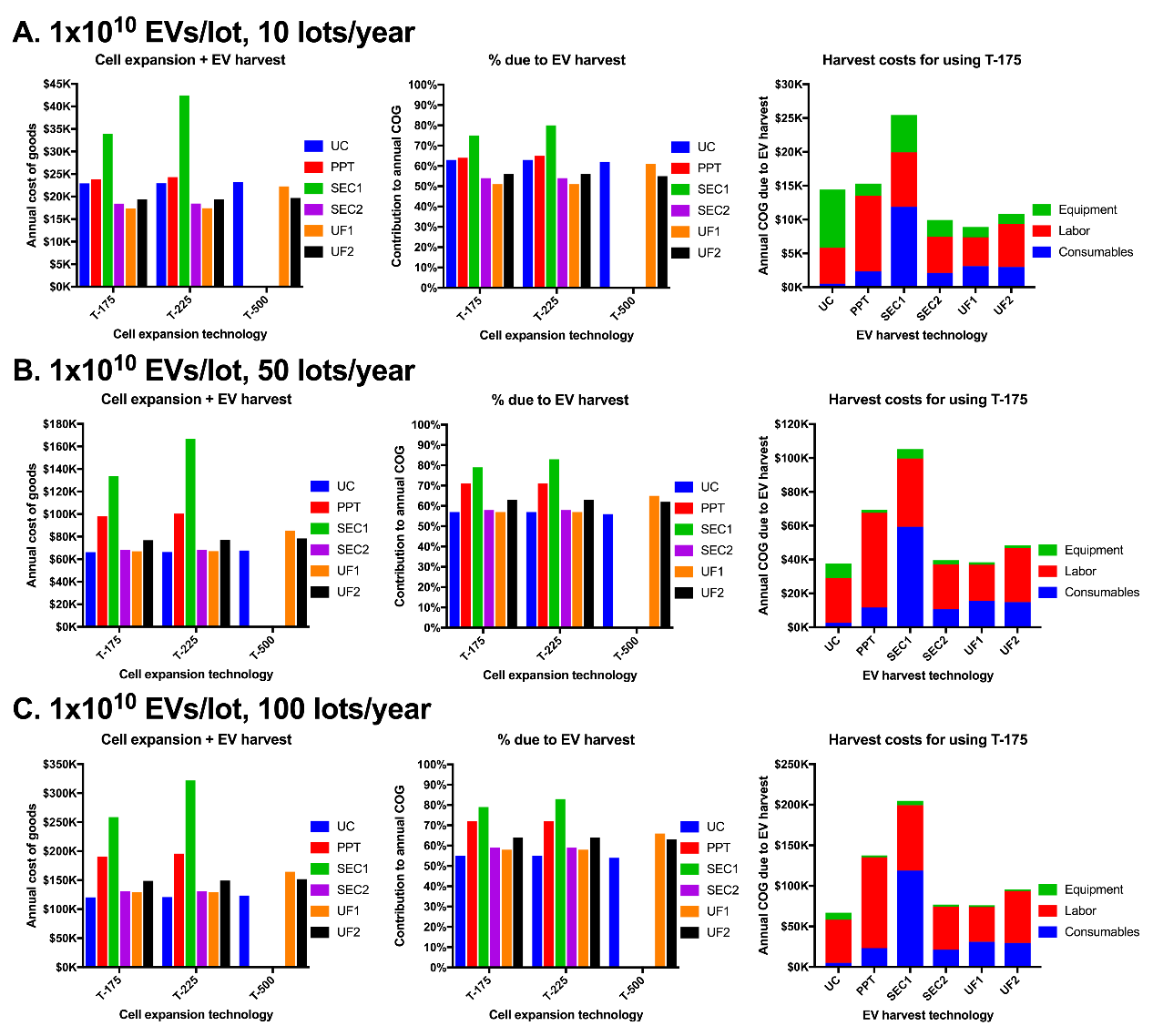


Supplementary Figure 4. (A-C) At bench scale, most EV harvest protocols are comparable in annual COG. EV harvest generally costs more than cell expansion in a given bioprocess. For a fixed lot size, varying annual demand does not drastically change the cost structure of EV harvest technologies except in the case of UC, thereby mostly preserving the ranking between technology combinations. Excluded technologies violate the limitations imposed.


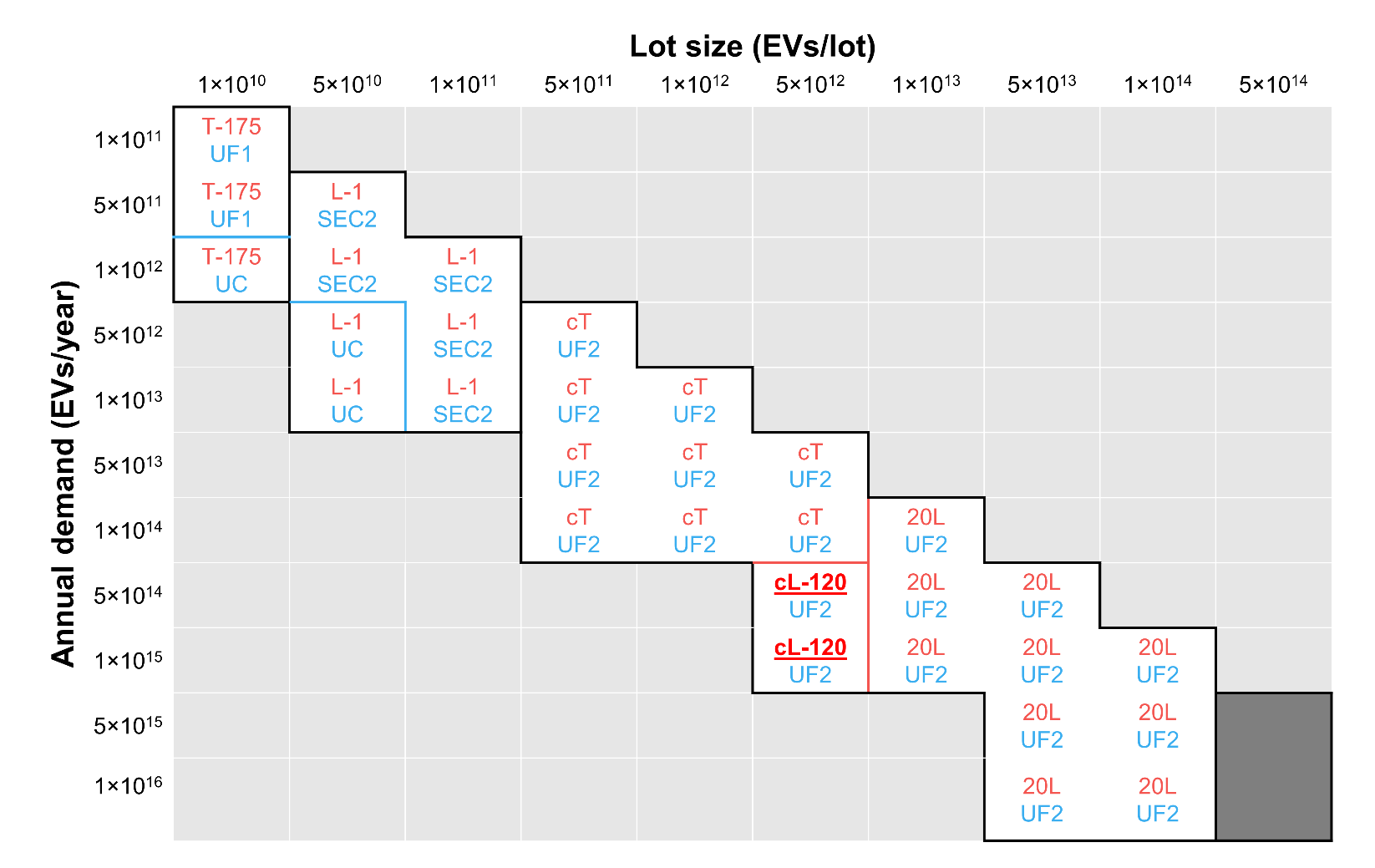


Supplementary Figure 5. Optimal technologies remain largely the same when labor rate is increased by 30%. Solutions that differ from those in **Figure 2C** are underlined and highlighted in dark blue. When labor rate is decreased by 30%, solutions remain identical to those in **Figure 2C**, except that UF1 is replaced by UC for 1×10^10^ EVs/lot at 5×10^11^ EVs/year.

Supplementary Figure 6: Example data showing relationship between cell and particle number. When cell and EV numbers from different donors and culture conditions were fitted into Equation 3, the average R2 was 0.83 +/- 0.081. See ‘Determination of Biological Parameters’ in the methods section for experimental details.

Supplementary Table 4. Key process and cost parameters.

| **Process parameter** |  |  | **Cost parameter** |  |
| --- | --- | --- | --- | --- |
| Seeding density ($d_{c}^{min}$) | 3000 cells/cm^2^ |  | Cell culture media ($p_{med}$) | $150/L |
| Maximum allowable density ($d_{c}^{max}$) | 25,000 cells/cm^2^ |  | Microcarriers ($p_{mc}$) | $5/g |
| Surface area of a microcarrier ($a_{mc}$) | 2930 cm^2^/g |  | Labor rate ($p_{lab}$) | $200/h |
| Microcarrier density ($d_{mc}$) | 6.3 g/L |  | Labor multiplier ($\beta$) | 2.2 |
|  |  |  | Biosafety cabinet, BSC ($p_{bsc}$) | $17,000 |
|  |  |  | BSC capacity ($U^{bsc}$) | 1 operator/BSC |
|  |  |  | Depreciation period ($t_{dep}$) | 10 years |

Supplementary Table 5. Process and cost parameters for cell expansion technologies.

|  |  |  | Media usage | |  | Labor (h) | |  |  |  |  |  |  |
| --- | --- | --- | --- | --- | --- | --- | --- | --- | --- | --- | --- | --- | --- |
| Cell expansion technology | Surface area (cm^2^) | Vessel price ($) | Planar (mL/cm^2^) | SUB (mL) | Operator capacity | Seed time | Collect time | Biosafety cabinet? | Incubator capacity | Incubator price ($) | Ancillary capacity | Ancillary price ($) | Maximum number of units per lot |
|  | $a_{i}^{pln}$ | $p_{i}^{vess}$ | $V_{i}^{pln}$ | $V_{i}^{sub}$ | $U_{E,i}^{m}$ | $t_{i}^{seed}$ | $t_{i}^{coll}$ | $\delta_{i}$ | $U_{i}^{inc}$ | $p_{i}^{inc}$ | $U_{i}^{anc}$ | $p_{i}^{anc}$ | $U_{E,i}$ |
| **T-flasks** | | | | | | | | | | | | | |
| T-175 | 175 | 9 | 0.25 | N.A. | 10 | 0.38 | 0.38 | 1 | 100 | 17,835 | N.A. | N.A. | 80 |
| T-225 | 225 | 10 | 0.25 | N.A. | 10 | 0.38 | 0.38 | 1 | 100 | 17,835 | N.A. | N.A. | 80 |
| T-500 | 500 | 15 | 0.40 | N.A. | 10 | 0.38 | 0.38 | 1 | 100 | 17,835 | N.A. | N.A. | 80 |
| **Multi-layers** | | | | | | | | | | | | | |
| L-1 | 636 | 60 | 0.25 | N.A. | 1 | 0.15 | 0.15 | 1 | 60 | 17,835 | N.A. | N.A. | 80 |
| L-2 | 1,272 | 73 | 0.25 | N.A. | 1 | 0.15 | 0.15 | 1 | 60 | 17,835 | N.A. | N.A. | 80 |
| L-5 | 3,180 | 241 | 0.25 | N.A. | 1 | 0.20 | 0.20 | 1 | 24 | 17,835 | N.A. | N.A. | 80 |
| L-10 | 6,360 | 507 | 0.25 | N.A. | 1 | 0.25 | 0.25 | 0 | 12 | 17,835 | N.A. | N.A. | 80 |
| L-40 | 25,440 | 1,265 | 0.25 | N.A. | 4 | 0.08 | 0.08 | 0 | 16 | 30,000 | 16 | 425,000 | 80 |
| **Compact flasks** | | | | | | | | | | | | | |
| cT | 1,720 | 19 | 0.33 | N.A. | 10 | 0.38 | 0.38 | 1 | 100 | 17,835 | N.A. | N.A. | 80 |
| **Compact multi-layers** | | | | | | | | | | | | | |
| cL-12 | 6,000 | 575 | 0.22 | N.A. | 1 | 0.20 | 0.20 | 0 | 24 | 17,835 | N.A. | N.A. | 80 |
| cL-36 | 18,000 | 1,050 | 0.22 | N.A. | 1 | 0.25 | 0.25 | 0 | 12 | 17,835 | N.A. | N.A. | 80 |
| cL-120 | 60,000 | 3,000 | 0.20 | N.A. | 4 | 0.08 | 0.08 | 0 | 16 | 30,000 | 16 | 425,000 | 80 |
| **Multi-layer bioreactors** | | | | | | | | | | | | | |
| bL-10 | 6,360 | 2,506 | 0.27 | N.A. | 1 | 0.75 | 0.25 | 0 | 6 | 17,835 | 1 | 56,000 | 80 |
| bL-50 | 31,800 | 5,586 | 0.19 | N.A. | 1 | 0.75 | 0.25 | 0 | 4 | 17,835 | 1 | 56,000 | 80 |
| bL-180 | 114,480 | 13,986 | 0.17 | N.A. | 1 | 0.75 | 0.25 | 0 | 2 | 17,835 | 1 | 56,000 | 80 |
| **Hollow-fiber bioreactors** | | | | | | | | | | | | | |
| HF | 21,000 | 12,000 | 0.37 | N.A. | 1 | 0.20 | 0.20 | 0 | N.A. | N.A. | 1 | 150,000 | 80 |
| **Microcarrier-based single-use bioreactors** | | | | | | | | | | | | | |
| 20L | N.A. | 2,000 | N.A. | 15 | 4 | 0.08 | 0.08 | 0 | N.A. | N.A. | 1 | 185,000 | 8 |
| 200L | N.A. | 4,500 | N.A. | 150 | 4 | 0.08 | 0.08 | 0 | N.A. | N.A. | 1 | 215,000 | 8 |
| 500L | N.A. | 5,850 | N.A. | 375 | 4 | 0.08 | 0.08 | 0 | N.A. | N.A. | 1 | 320,000 | 8 |
| 1000L | N.A. | 8,850 | N.A. | 750 | 4 | 0.08 | 0.08 | 0 | N.A. | N.A. | 1 | 425,000 | 8 |
| 2000L | N.A. | 10,500 | N.A. | 1,500 | 4 | 0.08 | 0.08 | 0 | N.A. | N.A. | 1 | 575,000 | 8 |
